# Functional Micro- and Nanofibers Obtained by Nonwoven Post-Modification

**DOI:** 10.3390/polym12051087

**Published:** 2020-05-10

**Authors:** Tomasz Kowalczyk

**Affiliations:** Institute of Fundamental Technological Research, Polish Academy of Sciences (IPPT PAN), Pawinskiego 5B, 02-106 Warsaw, Poland; tkowalcz@ippt.gov.pl

**Keywords:** nanofiber post-modification, functional nanofibers, tissue engineering

## Abstract

Micro- and nanofibers are historically-known materials that are continuously reinvented due to their valuable properties. They display promise for applications in many fields, from tissue engineering to catalysis or sensors. In the first application, micro- and nanofibers are mainly produced from a limited library of biomaterials with properties that need alteration before use. Post-modification is a very effective method for attaining on-demand features and functions of nonwovens. This review summarizes and presents methods of functionalization of nonwovens produced by electrostatic means. The reviewed modifications are grouped into physical methods, chemical modification, and mixed methods.

## 1. Introduction

Electrospinning (electrical spinning) is one of the most effective methods of nanomaterial production. It has a very high throughput, enabling the construction of materials of different types of polymers. Fragile polymers, drugs, or even living cells can be electrospun. The physical conditions of electrospinning have been established [1]. The technique dates back to 1600 and has been reinvented many times [2]. The recent wave of interest in electrospinning enabled the production of materials in many fields, ranging from tissue engineering to sensors and catalysis. The post-processing of electrospun micro- and nanofibers is a valuable method for introducing new properties or producing new materials with desired features. Many techniques for synthesizing different types of nanofibers are currently being developed. They provide alternative methods to single fluid electrospinning [3] with subsequent functionalization. Such methods include coaxial [4], modified coaxial [5], side-by-side [6], and tri-axial [7] electrospinning, and other multiple fluid processes [8]. Such methods of fabrication can lead to composite structures, e.g., core-shell [9], Janus [10], tri-layer core-shell [11], and other complex structures [12]. Such methods are especially valuable for industrial processing, where single-step processes are cheaper and facile in comparison to multi-step processes. Single fluid electrospinning followed by post-treatment is, on the other hand, a simple technique that can create new materials and properties without laborious procedures necessary to confine the process to a single step [13]. It aims to obtain the desired functions of structural fibers.

This review is based on the author’s 15 years of laboratory experience with on-demand electrospun nanofibers. As the main subject of the author’s works has been applications of nanofibers in biology and medicine, this review will mainly (but not only) deal with different types of scaffolds for tissue engineering, with some references to ceramic nanofibers. A vast amount of research represents nanofiber post-processing, so the author needed specified keys to select the presented material. The review is based on scientific recognition. For clarity, specific techniques are usually represented by two examples. The author omitted complicated, multiple-step post-modifications. The author have designed this review for newcomers in the electrospinning community, in order to give them well-established knowledge on the post-processing of electrospun micro- and nanofibers.

**Figure 1:**
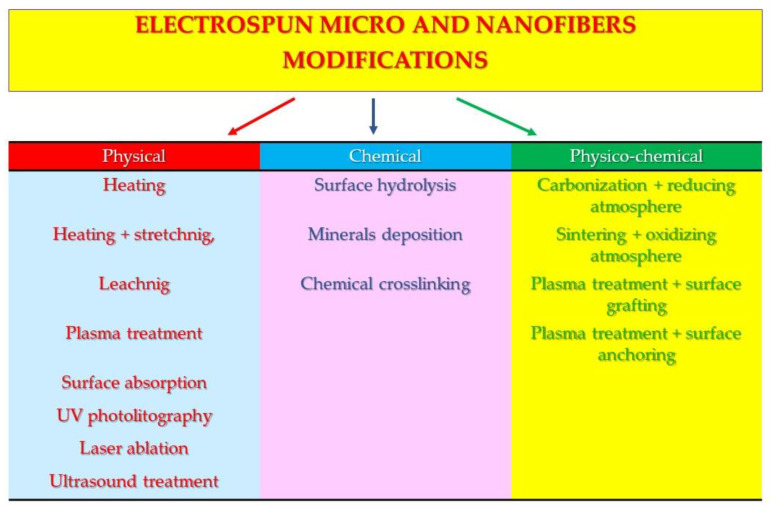
Scheme of reviewed modifications of micro- and nanofibers.

The chapters start with the simplest physical modification methods, followed by more complicated chemical modifications, and end with the most complicated physico-chemical modifications (Figure 1).

## 2. Physical Modifications of Micro- and Nanofibers

**Figure 2:**
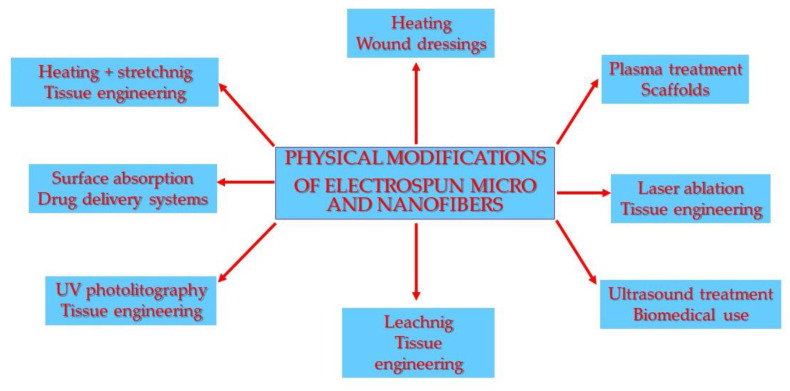
Scheme of physical modifications of micro- and nanofibers and their applications.

Physical surface modifications of electrospun nanofibers are directed towards thermally altering properties, stretching to change mechanical properties, the leaching or absorption of water-soluble polymers, plasma processing to increase the surface hydrophilicity, ultraviolet and laser ablation to produce patterned change of properties, and ultrasonic treatment directed towards increasing the porosity or fiber fragmentation (Figure 2) (Table 1).

### 2.1. Heating

One of the simplest physical methods that affect the properties of electrospun nanofibers is temperature curing. Kowalczyk et al. [14] cured bovine serum albumin (BSA) nonwoven, while Noszczyk et al. [15] cured micro- and nanofibers of human serum albumin (HSA). In both cases, nonwovens of protein and poly(ethylene oxide) (PEO) were not water-resistant. Their dissolution in water or phosphate buffer saline (PBS) was swift; storing them at human body temperature (37 °C) rendered them insoluble. Very soft conditions of fiber hardening were applied to protect biodegradable polymer matrix functions. Nonwovens hardened at human body temperature can be used as drug delivery systems for therapeutics (e.g., antimicrobials) that can survive matrix curing conditions. Sullivan et al. [16] electrospun the composition of poly(ethylene oxide) with whey protein or **β** -lactoglobulin (BLG) and used heat to render fibers insoluble in water. The fiber-forming PEO used was as low as 25%, and the mat was heated to 100 °C to make it insoluble in water. The mat swelled in water and retained a fibrous structure, which suggested its possible use in regenerative medicine. Rhodamine B (RhB), as a model flavonoid, was incorporated into the mat and subsequently released. Min et al. [17] electrospun regenerated silk fibroin (SF) and denatured the protein by steam treatment. The structure of fibroin changed from a random coil to a water-insoluble β sheet of a better mechanical strength. Human normal fibroblast and keratinocyte adhesion and a spreading study showed excellent cellular compatibility. The material is a candidate for wound dressing or scaffolds for tissue engineering. Enayati et al. [18] studied nonwoven composites of poly(vinyl alcohol) (PVA) with the addition of hydroxyapatite nanoparticles or cellulose nanofibers (CelluloseNF). The composites were heated to 180 °C to render them insoluble in water. The authors analyzed the influence of nano-components on the crystallinity of temperature-hardened poly(vinyl alcohol).

The thermal treatment of nonwovens and thermal matrix carbonization or degradation to form carbon or ceramic nanofibers is described in the chapter “Physico-chemical modifications.”

### 2.2. Heating and Stretching

**Figure 3:**
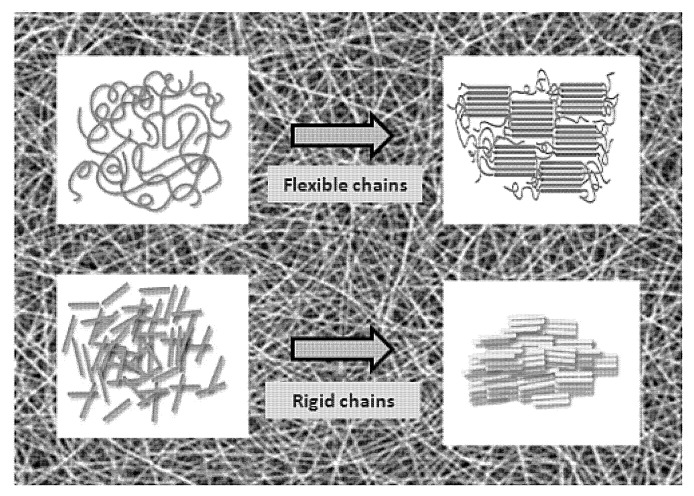
Schematic illustration of a polymer microdomain’s behavior after nonwoven mat stretching. Reproduced from [19] under the CC BY license. Copyright by MDPI.

Usually, as spun nanofibers have a low degree of molecular orientation, stretching can improve their crystallinity and strength. A generally higher degree of crystallinity accompanies a far lower elasticity and decreased rates of bio-degradation. The stretching of electrospun nanofibers is quite an unconventional method of modification. The strength and modulus results of single fibers are improved by up to two orders of magnitude when compared with nonwoven mats [19] (Figure 3).

Lai et al. [20] produced highly aligned micro- and nanofibers from poly(acrylonitrile) (PAN) copolymer. Subsequently, the authors stretched the nonwoven in steam so that it was up to four times the original length. As spun nanofibers were loosely oriented, even a small amount of stretching improved the orientation and degree of crystallinity. The authors attributed this to the zigzag conformation of the crystalline phase of stretched nanofibers compared to the helical one found in conventionally drawn microfibers. Steam-pulled fiber bundles had a tensile strength improvement of ca. 330% and Young’s modulus improvement of ca. 410%. Jundziłł et al. [21] found the appearance of two modes on the stress–strain curve of as-spun poly(L-lactide-*co*-caprolactone) (PLC) nonwoven used for tissue engineering. It suggested that pre-implantation stretching can drastically change the mechanical properties. Such treatment can improve tissue to implant matching. Zong et al. [22] uniaxially stretched electrospun nanofibers for heart patches. This post-processing of poly(lactide-*co*-glycolide) (PLGA) or poly(L-lactide) (PLLA) nanofibers led to anisotropic scaffolds used for cardiac cell cultures. Primary cardiomyocytes on poly(L-lactide) nanofibers developed functional contractile machinery (sarcomeres). They were electrically active.

Thermal treatment and nanofiber mat relaxation may be essential for preventing the nonwoven mat from shrinking after its withdrawal from a target. The treatment is necessary for polymers electrospun below the glass transition temperature (T_g_), e.g., poly(L-lactide-*co*-caprolactone) (PLC). PLC can be spun above T_g_, but for electrospinning at room temperature, additional thermal treatment might be necessary.

Wingert et al. [23] studied the relaxation time of poly(ω-undecanamide) (Nylon 11) electrospun nanofibers of T_g_ = 30–40 °C. Fong and Reneker [24] studied the phase separation of as-spun nanofibers of the styrene-butadiene-styrene (SBS) triblock copolymer that occurred upon annealing at 25 °C for 20 days. They observed the same effect at 70 °C annealing for 30 min. Polyvinylidene fluoride (PVDF) nanofibers were heated at 150–160 °C by Choi et al. [25] to consolidate them on a membrane with a lower porosity and higher crystallinity. The thermal treatment sharply improved the tensile strength, elongation at break, and tensile modulus. Liao et al. [26] used a hot press at 170 °C, just above the melting point of nonwoven PVDF, to mechanically consolidate the nonwovens for use in direct contact membrane distillation. The authors found that heat-press post-treatment improved the membrane mechanical integrity, enhanced water permeation, and prevented membrane pores from wetting during direct contact membrane distillation. The resulting membranes had better properties than commercial PVDF membranes or their counterparts electrospun with added clay.

### 2.3. Leaching

Water-soluble polymers, e.g., poly(ethylene oxide) or poly(vinyl alcohol), are commonly used to improve the spinnability. They are also used as a fiber-forming polymer by mixing with non-spinnable polymers. These synthetic polymers, as well as natural ones, can be leached out of composite nanofibers to create nanoporosity. Poly(vinyl alcohol) (PVA) is highly hydrophilic, and draining it out of the polymer matrix may be challenging.

Zhang et al. [27] studied the electrospinning of poly(caprolactone) (PCL) with gelatin. The protein leaching led to 3D porous scaffolds for tissue engineering. Soaking a nonwoven in a phosphate buffer saline at 37 °C produced a nano-topography with grooves, ridges, and elliptical pores. The BET surface area was 2.4 times bigger after leaching the gelatin. The material is a candidate for tissue engineering and industrial applications. Poly(caprolactone) electrospun from volatile solvents at mild temperatures and a high humidity forms a similar structure. Bhattarai et al. [28] electrospun low molecular weight poly(ethylene oxide) (PEO) mixed with poly(L-lactide) (PLLA). The nonwoven gradually leached PEO when placed in phosphate buffer saline at 37 °C. The composition with 50% PEO showed about 80% burst leaching; while the composition containing 20% gradually leached PEO for up to six weeks. Fibroblasts seeded on the scaffolds with 20% PEO showed better cell–matrix interactions and cell morphologies compared with 50% PEO or pristine PLLA nanofibers. Mozafari et al. [29] electrospun poly(vinyl alcohol) with chitosan to form composite nanofibers for neural tissue engineering. PVA gradually leached from the scaffolds within a week. Embryonic cells from a neural crest seeded on the scaffolds showed enhanced viability and proliferation when compared to the control.

### 2.4. Surface Absorption

A protein coating on scaffolds can increase the capacity of the cells to recognize the surface. Absorbed proteins can play a role as amphiphilic substances temporarily increasing the (bio) availability of the scaffold surface. It is one of the simplest methods available, needs no medical approval for use, and has no adverse effect on the mechanical properties of a polymer matrix. The technique is, therefore, especially valuable, even if the results are not as good as those obtained for permanently modified scaffolds.

Zhang et al. [30] soaked poly(caprolactone) nanofibers in a diluted collagen solution overnight. The authors compared the proliferation and cell morphology of human dermal fibroblasts seeded on scaffolds immersed in collagen and collagen spun with poly(caprolactone). The cellular behavior of arduously created co-electrospun nonwovens was compared with those made by the general electrospinning procedure and soaking in protein. The latter matched the effect, with about a two-day longer cell incubation time. Studied scaffolds are designed for tissue engineering. Koh et al. [31] produced scaffolds for neural tissue engineering by the physical attachment of laminin to electrospun PLLA nanofibers. The authors found that this protein bonded to nonwoven promoted neurites. Embryonic cells from a neural crest (PC12) displayed enhancement in neural extensions that were less extensive for physically absorbed laminin and covalent bonding than for blend polymer electrospinning. However, the last two methods needed far more effort and did not guarantee a native form of laminin. Venugopal et al. [32] produced a dermal substitute from nonwoven poly(caprolactone) (PCL), using collagen to facilitate interactions between cells and the scaffold. Human dermal fibroblasts showed better attachment and growth on nanofibers with physically attached collagen, compared to pristine PCL nonwovens. Synthetic nonwovens were inferior to electrospun natural collagen in terms of the cellular biocompatibility, yet they had much better mechanical properties and a longer degradation time. Lu et al. [33] used a coaxial technique to produce coaxial micro- and nanofibers of poly(caprolactone) (core) and cationized gelatin (shell). The nonwovens were crosslinked with glutaraldehyde to serve as a drug carrier. The microfibrous gel was immersed in solutions of two conjugates of protein with fluorescein isothiocyanate—BSA-FITC, heparin-FITC, and in vascular endothelial growth factor (VEGF)—for drug delivery assessment. Initial concentrations of protein-absorbed in nonwovens varied from 0.12% to 0.23% relative to the mass of the membrane. The authors assessed the release of VEGF for 15 days. Lee et al. [34] produced electrospun nonwovens from poly(lactide-*co*-glycolide) as scaffolds for guided bone regeneration. They coated fibers by soaking them in poly(dopamine). Bone-forming protein −1 (BFP1) was introduced to nonwovens pre-coated with poly(dopamine) (PD) by incubating them overnight at 37 °C. Human mesenchymal stem cells seeded on the scaffolds formed an implant for the mouse calvarial defect model. The implants showed 2–3 times more bone regeneration after two months *in vivo* when compared with a non-implant group. A very appealing approach was presented by Zhang et al. [35], who covered poly(caprolactone) nonwovens with hydrophobins. These fungal proteins contain hydrophilic and hydrophobic regions. Anti-CD31—an antibody specific for endothelial cells—was bonded by protein–protein interactions with the hydrophobin layer. This covered nonwoven promoted the attachment and retention of endothelial cells. However, this elegant and general approach has not found many followers in the electrospinning community. Zhou et al. [36] covered cellulose nanofibers with negatively charged gold nanoparticles and positively charged lysosome using the layer-by-layer self-assembly technique. The composites produced exhibited excellent antibacterial activity against *E. coli* and *S. aureus*.

### 2.5. Plasma Treatment

Plasma treatment is a very convenient method, and is mainly used to increase the surface hydrophilicity. It is widely used in industry to modify polymer films [37]. The main parameters of plasma modification are the type of gas (e.g., Ar or O_2_), power, and treatment time. Plasma-treated polymers required further processing. The surface effects of plasma modification are only temporary and are gradually lost over time.

Poly(caprolactone) nanofiber meshes have been modified by radio-frequency (RF) plasma to enhance cell adhesion, differentiation, and proliferation. For fibroblasts, osteoblasts, and chondrocytes, oxygen plasma treatment was the most successful approach. It showed the greatest enhancement of the contact angle and material–cell interactions. The authors also noted that the surface roughness is a key factor in material–cell interactions [38]. Electrospun poly(caprolactam) (PA6) treated with oxygen plasma for 1 min showed an increase in the oxygen content, enhancement of the contact angle, and a surface roughness smaller than the sample treated for 5 min [39]. Silk fibroin nanofibers have been modified by CH_4_ or oxygen plasma to produce scaffolds for skin regeneration. Methane plasma modification slightly influenced the hydrophilicity compared to oxygen plasma. The latter caused a large increase in the wettability and cellular activity of human epidermal keratinocytes and fibroblasts [40]. Ammonia or oxygen plasma substantially altered the surface composition and hydrophilicity of micro- and nanofibers of poly(lactide-*co-*glycolide). The adhesion and proliferation of mouse fibroblasts were also positively affected. The degradation, in contrast, sped up, making the material less favorable for the production of scaffolds for tissue engineering [41]. A short plasma treatment of poly(L-lactide) micro- and nanofibers increased their surface oxygen content and significantly reduced the water contact angle. The treated nonwovens displayed an initial enhancement of porcine mesenchymal stem cell adhesion and better morphology, which could be useful for scaffolds for tissue engineering [42]. Glow discharges combined with gas mixtures of N_2_ + H_2_, NH_3_ + O_2_, and Ar + O_2_, increased the hydrophilicity of nonwovens. Mouse fibroblasts seeded on plasma-modified poly(caprolactone) nanofibers gave higher proliferation and adhesion rates compared to untreated fibers. Plasma modification only had a limited negative impact on the material tensile properties [43]. De Valence et al. [44] very elegantly linearized the time of plasma exposure vs. the change of the contact angle. Poly(caprolactone) nonwovens modified for 0–60 s are useful for tailoring the properties of nanofibers. Hydrophilized scaffolds seeded with smooth muscle cells were subcutaneously implanted as an aortic replacement in a rat model for a three-week follow-up period. Even for the entirely hydrophilic nonwoven, the fiber morphology and mechanical properties were left intact. Cells on treated scaffolds had a spread-out morphology, while on untreated nonwovens, the cells were small and rounded. Subcutaneous implantation revealed a low foreign body reaction. Implants of modified nonwovens and cells were used as an aortic replacement. They caused better cellularization of the graft wall, and the level of endothelization was left intact. The authors found the optimal conditions for modifying the surface of nanofibers whilst leaving the mechanical properties unchanged and produced biomaterial with features making it valuable as a vascular scaffold.

### 2.6. UV Photolithography and Laser Ablation

UV or laser beams can be applied for the patterning and controllable surface modification of electrospun nanofibers. These methods of selectively changing the surface properties can create a pattern of “well-like” 2D environments for directed cell culturing.

Yixiang et al. [45] studied UV degradation and photolithography on poly(D,L-lactide-*co*-glycolide) (PDLG) and poly(L-lactide-*co*-caprolactone) (PLC) nanofibers. The authors used a commercial germicide sterilization UV lamp (wavelength 254 nm). One hour of irradiation led to a ca. 40% decrease in the molecular weight and ca. 30% decrease in the tensile strength. Masked UV irradiation produced patterned nonwovens. Smooth muscle cells migrated to irradiated wells. Lim et al. [46] used a femtosecond laser to ablate patterns on electrospun poly(caprolactone)/gelatin nonwovens. Mouse embryonic stem cells were seeded on the scaffolds to assess the cell density. A comparison of one and two days of culturing showed no difference between ablated and unmodified fibers. Cells confined to the ablated wells were less dense than on pristine nonwoven on the third day of culture. Lee et al. [47] showed a similar approach, using a femtosecond laser to create a cell-friendly pattern on the surface of nonwoven poly(L-lactide). Laser ablation led to holes with diameters of 50, 100, and 200 micrometers, and prefixed spacing. Cells seeded on ablated scaffolds had different morphologies, but the same proliferation, as those on non-ablated nonwovens. Animal studies have shown that patterned nanofibers facilitate endothelial cell growth and drastically increase cell infiltration. Such scaffolds are useful for tissue engineering.

### 2.7. Ultrasonic Treatment

Ultrasonic treatment leads to the degradation of polymer chains. Its use in polymer nanofiber treatment is therefore limited to surface treatment and fiber scission. Scaffolds with pores enhanced by sonification have a cotton candy structure of lower mechanical properties; the polymer matrix is also pre-degraded.

Lee et al. [48] used ultrasonication to increase the porosity and pore size of electrospun nonwoven poly(L-lactide) and poly(caprolactone). The treatment greatly enlarged the porosity, pore size, and amount of fibroblasts seeded on the scaffolds. It also significantly increased the cell infiltration potential, leading to real 3D scaffolds for tissue engineering. Gu et al. [49] ultrasonicated chitosan nonwovens to produce hemostatic material. During treatment, the porosity of the membrane increased from 80% to 97%, and the water absorption time decreased from 110 to 9 s. The blood clotting efficiency of ultrasonicated chitosan nonwovens was ca. 1.3 to 3.4 times better than that for commercial Surgicel and a chitosan sponge. The human dermal fibroblast culture displayed a 1.4 times better proliferation rate on ultrasonicated chitosan nonwovens compared to the pristine one. Electrospun nanofibers were scissored onto short fibers by ultrasonic treatment by Sawawi et al. [50]. Ultrasound cavity bubble implosion caused the effect. Brittle poly(styrene) (PS) and poly(methyl methacrylate) (PMMA) membranes readily produced ca. 10 micrometer short fibers. More flexible polymers, such as poly(L-lactide) or poly(acrylonitrile), required pre-processing before ultrasonic scission.

## 3. Chemical Modification of Micro- and Nanofibers

**Figure 4:**
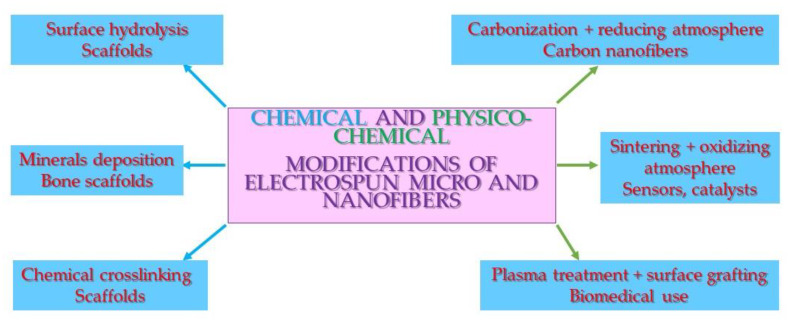
Scheme of the chemical and physico-chemical modifications of micro- and nanofibers and their applications.

Chemical modifications of nonwovens are far more complicated than physical ones. The main applications are stable surface modification, the crosslinking of water-soluble nonwovens, and precipitating mineral compounds on the surface. The main drawbacks are the need for the use of non-medically approved chemicals and the speeding up of polymer matrix degradation (Figure 4 and Table 2).

### 3.1. Surface Hydrolysis

Wang et al. [51] produced hyaluronic acid (HA) nonwovens by blowing assisted electrospinning. The authors conducted multi parameter optimization. The goal was to produce water-resistant HA membranes with a reasonable yield. Maintaining the nanofibrous structure was also necessary. The authors proposed a unique, mild-condition, non-toxic crosslinking protocol employing hydrochloric acid and attained a consistent quality electrospun HA membrane.

Surface hydrolysis is a standard procedure used to increase the hydrophilicity of polymer scaffolds. It helps cell attachment, proliferation, and differentiation. The process is pronounced not only on the surface, but also in the bulk polymer. It speeds up the degradation of polymer nanofibers.

Boland et al. [52] used hydrochloric acid hydrolysis to improve poly(glycolide) (PGA) nanofibers’ soft tissue biocompatibility. Rat cardiac fibroblasts seeded on hydrolyzed scaffolds enabled proliferation even better than the tissue plastic control. In contrast, pristine nonwovens exhibited a low rate of cell proliferation. Polymer-cell constructs were implanted intramuscularly. Pristine nonwoven cell implants caused fibrous encapsulation in vivo; in contrast, nanofiber hydrolysis led to cellular implants being well-incorporated into the surrounding tissue. Park et al. [53] used a sodium hydroxide solution to modify poly(caprolactone) nonwovens to promote osteoblast adhesion and proliferation. The nanofibers retained their shape and diameter. Hydrolyzed scaffolds formed a favorable environment for cells to proliferate and metabolize compared to unmodified fibers. Gao et al. [54] used alkaline hydrolysis to modify poly(glycolide) nanofibers, in order to increase the cell seeding density and improve the attachment of vascular smooth muscle cells. The nonwoven maintained its dimensions and thermal properties, while the fiber diameter decreased after hydrolysis with dilute sodium hydroxide solution. The authors linearized the decrease of fibers’ diameter versus the hydrolysis time. More than twice the amount of cells colonized the hydrolyzed nonwoven compared to the pristine one. Individual cells were attached to modified material, while only cell aggregates appeared on control nonwovens.

### 3.2. Mineral Deposition

Hydroxyapatite (HAP) deposition is an alternative method to direct addition of HAP nanoparticles to an electrospun solution. It creates biocompatible minerals on the surface of micro- and nanofibers, at places where cells will recognize them. This method is mainly used for bone tissue engineering.

**Figure 5:**
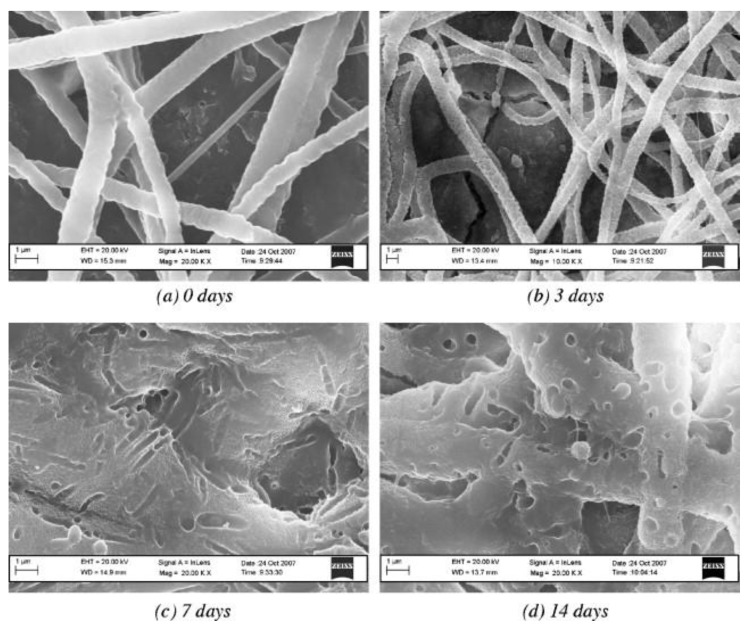
SEM images of a poly(caprolactone) (PCL)–poly(ethylene oxide) (PEO) composite fibrous coating on a Bioglass^®^ sintered pellet after immersion in SBF for the specified number of days. Reproduced with permission from [55]. Copyright by Elsevier.

Bretcanu et al. [55] used biocompatible Bioglass pellets as models of ready-to-use implants. Tiny electrospun nonwovens of poly(caprolactone) or poly(hydroxybutyrate-*co*-hydroxyvalerate) (PHBV) enclosed Bioglass. Materials were covered with HAP from SBF and displayed electrospun nanofiber degradation, which was the least extensive on PCL nanofibers (Figure 5). Chen et al. [56] tested different methods of precipitation of bone-like hydroxyapatite (HAP) on nanofibers to prepare scaffolds for bone implants. The authors found that pristine poly(L-lactide) nanofibers exhibited a reasonable degree of HAP precipitation from simulated body fluid (SBF). Some addition of citric acid or poly(L-aspartic acid) almost completely inhibited the precipitation. A short period of alkaline etching sped up the deposition by ca. 50%. Meng et al. [57] produced mineralized surface scaffolds for bone tissue engineering. The authors used simulated body fluid, the supersaturated calcification method, and an alternative soaking method. The surface containing gelatin led to the formation of a more substantial amount of apatite compared to pristine poly(D,L-lactide-*co*-glycolide) nanofibers. Human osteosarcoma cell lines exhibited an excellent biocompatibility for the scaffolds. Cell adhesion, proliferation, and differentiation were reasonable for all mineral layers produced.

### 3.3. Chemical Crosslinking

Water-soluble, bioactive polymers can be electrospun in a native bio-recognized form. The crosslinking of such nonwovens makes them insoluble and useful as scaffolds for tissue engineering.

Chen et al. [58] crosslinked electrospun gelatin nonwovens with genipin for peripheral nerve conduits. The material was subcutaneously implanted in a rat model, caused a mild tissue response, and only formed a thin fibrous capsule. The rat sciatic nerve gap was repaired with the conduit with full regeneration after 4, 6, and 8 weeks. Numerous regenerated nerve fibers reconnected through the gap to produce adequate nerve functional recovery. Zhang et al. [59] studied optimization of the gelatin exposure time to glutaraldehyde gas. The goals were the best mechanical properties and cell response. Crosslinking also led to an improvement of the tensile strength by ten times and denaturation temperature by 11 °C compared to pristine gelatin nanofibers. Human dermal fibroblasts demonstrated a linear increase in cell density over time while seeded on crosslinked gelatin scaffolds. Residual glutaraldehyde caused only a small extent of initial inhibition due to its cytotoxicity. Another work on the crosslinking of gelatin type A or B nanofibers with gaseous glutaraldehyde provided by Ratanavaraporn et al. [60] gave the best results when compared with different physical methods. Spraying or immersion with 1-ethyl-3-(3-dimethylamino propyl) carbodiimide hydrochloride (EDAC) solution led to swollen fibers. Glutaraldehyde gas produced a merged nanofiber structure.

## 4. Physico-Chemical Modifications of Micro- and Nanofibers

Physico-chemical modification is the most frequently used method of nonwoven post-processing. It allows the fabrication of nanomaterials of entirely new, non-spinnable materials (graphite nanofibers and ceramic nanofibers), surface grafting with polymers, and the stable anchoring of biological molecules on the surface of nanofibers (Figure 3) (Table 3).

### 4.1. Carbonization in Reducing Atmosphere

**Figure 6:**
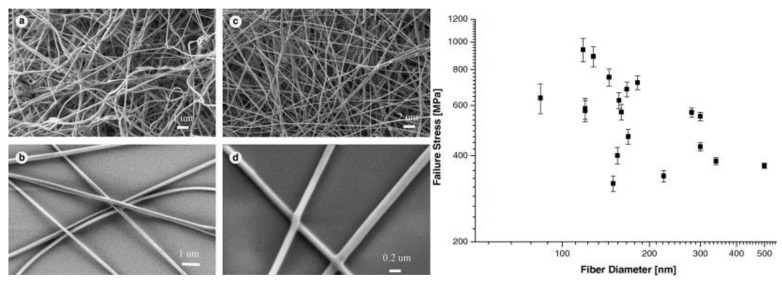
(**a**) and (**b**) show SEM images of electrospun nanofibers and (**c**) and (**d**) show SEM images of carbonized nanofibers. Plot of carbon fiber failure stress as a function of the fiber diameter. Reproduced with permission from Elsevier [61].

Zussman et al. [61] studied the carbonization of electrospun poly(acrylonitrile) nanofibers. The authors assessed the mechanical properties and structure of graphitized carbon nonwovens. They found 20% of remaining sp^3^ bonds and ca. 10.5% of non-carbon atoms in the nanofibers. The micromechanical testing of numerous individual fibers gave an average modulus of 63 GPa and a diameter of 50–250 nm (Figure 6.). Carbon nanofibers are primarily produced by the oxygen-free carbonization of electrospun polymers (90% from poly(acrylonitrile) [62]). Ra et al. [63] turned electrospun poly(acrylonitrile) onto carbon nanofibers by the single-step method. The nonwoven was placed in an oven and stabilized in the air up to 280 °C, and after heating to 700–1000 °C, the atmosphere switched to argon. The carbon paper produced had significantly better capacitance and energy density retention than activated carbon. The material was designed for the production of high power supercapacitors. Kim et al. [64] compared two types of graphitization of poly(acrylonitrile) electrospun nonwovens. Samples graphitized at 700 °C had a ca. 350 times lower conductivity than those graphitized at 1000 °C. The crystallite size was twice as big for the latter carbon nanofibers.

### 4.2. Sintering in Oxidizing Atmosphere

The most common method employed for the production of ceramic nonwovens is thermal sintering. The process conditions, mainly the temperature, gas atmosphere, and sintering time, determine the nature of produced materials. Metal oxide nanofibers are produced from nonwoven precursors in the oxidative atmosphere and used as sensors or catalysts (catalyst beds). Most frequently, metal oxide precursors do not form fibers. Spinnable polymers, such as poly(vinyl pyrrolidone) (PVP), poly(vinyl alcohol), and poly(acrylonitrile), are added to form nanofibers. The polymer matrix is subsequently burned out in the oxygenated atmosphere, while metal oxide particles are sintered.

Nanofibers of titanium dioxide nanoparticles suspended in poly(vinyl acetate) were deposited onto arrays of platinum electrodes, pressed to 120 °C, and calcinated at 450 °C. The material formed nanometer-size anatase crystals and showed an eight-fold increase in sensor resistance when used as a nitrogen dioxide sensor [65]. Titanium dioxide (anatase) nanofibers containing platinum nanoparticles were obtained by calcination of the nonwoven in the air at 500 °C. The mat was electrospun from poly(vinyl pyrrolidone) solution contained titanium tetraisopropoxide and platinum acetate. A comparison with pristine anatase nanofibers showed significant enhancement by the presence of Pt nanoparticles for hydrazine sensing in water samples [66]. Choi at al. [67] compared methods of the production and structure of nanofibers with their efficacy. Titania nanofibers were produced by the electrospinning of titania nanoparticles dispersed in poly(acrylonitrile) matrix that was subsequently burned out. Titania nanoparticles were sintered for comparison. Nanofibers exhibited three-times higher light energy conversion compared with nanofibers produced from nanoparticles. The authors attributed this phenomenon to mesoporosity and nanoparticle alignment that facilitated charge transfer. Formo et al. [68] electrospun titanium tetraisopopoxide in poly(vinyl pyrrolidone) matrix. The nonwoven was calcinated in the air at 510 °C. The anatase produced was covered with platinum nanoparticles by soaking the material in a polyol reduction bath. The material produced displayed excellent catalytic activity towards hydrogenation of the azo bond. Reddy et al. [69] covered titanium dioxide nanofibers with silver nanoparticles by soaking them in a silver salt-potassium citrate reducing bath. The catalytic bed produced had photocatalytic activity higher than that of pristine titania nanofibers. Titania nanofibers hydrothermally doped with tin dioxide exhibited high photocatalytic activity toward model dye (Rhodamine B). Tin dioxide nanofibers with nickel (II) oxide produced by electrospinning served as a humidity sensor [70]. Sensors made of the same materials showed a higher formaldehyde sensitivity compared to pure tin dioxide nanofibers. The effect was attributed to surface distortion and the different grain sizes of the catalyst [71]. Tin dioxide [72] or tin dioxide-zinc oxide [73] nanofibers were tested as ethanol sensors with a wide range, high response, and excellent linearity. Tin dioxide nanofibers produced from electrospun precursors were studied as hydrogen sulfide sensors. A comparison of bare tin dioxide nanofibers and those loaded with a minimal amount of copper (II) oxide showed an enhanced response, recovery time, and selectivity. The effect was attributed to the formation of p-n junctions due to the micro-grains formed [74]. Nanofibers of tin dioxide, compared with those containing a heavy load of palladium, were tested as hydrogen or nitrogen dioxide sensors. Nanofibers with up to 40 mol% of Pd were found to have a four orders of magnitude higher sensitivity, with a detection limit of several ppb. The effect was attributed to grain growth inhibition and the presence of catalyst enhancing oxidation [75]. Electrospun tin dioxide nanofibers formed a hydrogen sensor. A comparison of two types of nanofibers revealed that hollow nanofibers had the highest response compared to filled ones [76]. Nickel (II) oxide nanofibers containing platinum, compared with undoped nanofibers, showed considerable improvement of the electrocatalytic activity towards glucose detection. Doped nanofibers had a higher sensitivity, lower detection limits, and a good linear range [77]. An example of zirconia-titania nanofibers was used as a humidity sensor with an excellent characteristic. The impedance of the sensor changed by four orders of magnitude between very dry and very humid conditions [78]. Silica nanofibers synthesized from silicon tetraethoxylate, poly [3-(trimethoxysily)propyl methacrylate], and silver nitrate formed precursors to produce organosilicon fibers. Fibers were sintered to silica fibers containing silver nanoparticles. Their catalytic activity was assessed by methylene blue reduction [79].

A comprehensive review of ceramic nanofiber synthesis and application is given by Panda [80].

### 4.3. Plasma Treatment and Surface Grafting

Plasma treatment was used to induce surface-functionalized groups. They were further used for the chemical binding of biomolecules or grafting with hydrophilic polymers by free-radical polymerization.

Nanofibers produced from hydrophobic poly(glycolide), poly(L-lactide), and poly(lactide-*co*-glycolide) were subjected to oxygen plasma and in situ grafted with hydrophilic acrylic acid. Highly porous nonwovens with a surface-bonded poly(acrylic acid) moiety containing carboxylic groups had lower contact angles. Fibroblasts seeded on plasma-grafted nonwovens exhibited more significant attachment and proliferation when compared to unmodified nanofibers [81]. Similarly, poly(caprolactone) nanofibers pretreated in argon plasma were either surface oxidized or grafted with acrylic acid vapor. Oxidized and pristine fibers had a similar influence on the proliferation, differentiation, and viability of preosteoblast cells. Acrylic acid grafted nanofibers gave much better results and were suggested as scaffolds for bone tissue engineering [82]. The plasma treatment of micro- and nanofibers of poly(L-lactide) was combined with cationized gelatin grafting. Surface carboxyl groups produced by plasma were chemically bonded to gelatin amine groups by carbodiimide (CDI) coupling. The viability, proliferation, and differentiation of rabbit articular chondrocytes were better on grafted nonwovens compared to those which were unmodified. Chondrocytes were grown on a modified scaffold and maintained its phenotype. Animal studies of subcutaneous implants revealed the presence of ectopic cartilage after four weeks [83]. Electrospun poly(caprolactone) nanofibers were modified by remote plasma treatment, followed by type I collagen coating. The wettability, primary human dermal fibroblast attachment, spreading, and proliferation were enhanced in treated nanofibers compared to pristine ones. Remote plasma treatment was found to be more effective than conventional plasma [84]. Nonwoven poly(L-lactide) nanofibers were modified by plasma activation, followed by arginylglycylaspartic acid (RGD) (tripeptide Arg-Gly-Asp) coupling by 1-ethyl-3-(3-dimethylaminopropyl) carbodiimide hydrochloride (EDAC)-N-hydroxysulfosuccinimide (sulfo-NHS) activation. Oxygen plasma influenced the mechanical properties and reduced the hydrophobicity. The culturing of human mesenchymal stem cells in vitro on RGD-coupled nanofibrous scaffolds induced osteoinductive properties, but no difference in the proliferation or cell density was found [85].

The chemical bonding of proteins and biocompatible compounds is another step towards the biomimicry of an artificial scaffold used for tissue engineering.

Zhu et al. [86] fabricated an esophageal scaffold with improved epithelial tissue regeneration. They electrospun poly(L-lactide-*co*-caprolactone). The nonwoven was subjected to fast ammonolysis with 1.6-hexanediamine, followed by glutaraldehyde coupling and the covalent bonding of fibronectin to the spacer. The material strain decreased during the grafting, but the tensile strength remained unchanged. Porcine esophageal epithelial cells seeded on the modified scaffold displayed a proper phenotype and were much prominent when compared with the pristine one. Ghasemi-Mobarakeh et al. [87] chemically bonded Matrigel to a poly(caprolactone) nonwoven to produce a substrate for nerve tissue engineering. At first, alkaline hydrolysis created some carboxylate groups; subsequently, EDAC treatment was followed by Matrigel covalent bonding. Nerve precursor cells seeded on the scaffolds showed the best proliferation and neurite outgrowth on the Matrigel-modified scaffolds compared to pristine nanofibers and alkaline etched ones. Zhu et al. [88] used UV light to create free radicals on the electrospun PCL surface. Subsequent methacrylic acid grafting and EDAC treatment were used to covalently bond gelatin. The endothelial cell culture performed slightly better on the electrospun gelatin-modified scaffold than on the pristine one and control polymer membrane modified with poly(methacrylic acid) or gelatin. Kim and Park [89] electrospun a blend of poly(caprolactone) with specially synthesized block copolymer: poly(D,L-lactide-*co*-glycolide)-poly(ethylene oxide)-NH_2_. The particular copolymer was terminated with the amino group used to couple the lysosome. The enzyme was covalently linked using ethylene glycol-bis (sulfosuccinimidylsuccinate). The proposed methodology can be used to fabricate nanofibers with other bioactive molecules attached to the surface.

## 5. General Conclusions

Numerous reputable review articles of nonwoven post-modification are available. They concern specific fields of the vast subject of electrospun nonwoven modification, including the surface functionalization of electrospun nanofibers for tissue engineering and drug delivery [90]; tissue engineering [91]; architecture and fabrication for tissue engineering [92]; manufacturing, biofunctionalization, and cell interactions [93]; the use of free-radical methods of nonwoven modification [94]; the biological and chemical functionalization of electrospun scaffolds for cardiac tissue engineering [95]; the potential of nanofibers as matrices for tissue engineering [96]; tubular vascular grafts [97]; and scaffolds for medical applications [98].

As exemplified in Feynman’s famous statement—“there is plenty of the room at the bottom,”—there are a multitude of applications where nanofibers can be especially valuable. The technique was a curiosity when discovered; now, it is one of the most efficient and cheap ways to produce nanomaterials. The field of the use of such materials is far from being limited to the tissue engineering, wound dressing, sensors, and catalysis that the author has presented in this review. Being included in wearable electronics, power generation (wearable and external), telemedicine sensors, filtration and separation, health protection, and environmental remediation, the materials are also a part of the circular economy. The multitude of parameters governing electrospinning may seem scary, but they create unique possibilities to make any new material on demand. If the material is not good enough, one can use a multitude of surface modification techniques. Based on the steadily growing number of science articles that are discovering innovative materials and applications based on electrospinning and other ways of producing nanofibers (e.g., blow spinning), it is one of the new techniques that will change the world in the 21st century.

## Figures and Tables

**Table 1:** Methods of physical modifications of micro- and nanofiber nonwovens and their applications.

Polymer	Method of Modification	Application	Reference
**BSA-PEO (85-15)**	Heating 37 °C, 3–4 weeks	Biosensors	[14]
**HSA-PEO (50:50)**	Heating 37 °C, 3–4 weeks	Antiadhesive wound dressings	[15]
**WP-PEO RhB BLG-PEO**	Heating 100 °C	Regenerative medicine	[16]
**Silk fibroin**	Heating: steam 100 °C	Wound dressings, scaffolds for TE	[17]
**PVA/HAP, CelluloseNF**	Heating 180 °C	-	[18]
**PAN**	Heating: steam 100 °C, stretching up to 400%	High mechanical strength nonwovens	[20]
**PLLA, PLGA**	Heating 60 °C, uniaxial stretching 200%	Heart TE	[22]
**SBS**	Heating 25 °C, 20 days or 70 °C, 30 min	Nanometer range nanofibers	[24]
**PVDF**	Heating 150–160 °C	Polymer electrolyte or separator	[25]
**PVDF**	Heating 170 °C, 1 h, press	Direct contact membrane distillation	[26]
**PCL/Gelatin**	Heating in 37 °C PBS aq. bath, leaching	TE	[27]
**PLLA/PEO**	Heating in 37 °C PBS aq. bath, leaching	TE	[28]
**Chitosan/PVA**	Heating in 37 °C bath, leaching	Neural TE	[29]
**PCL**	Soaking in the collagen solution	Scaffolds for TE	[30]
**PLLA**	Physical attachment of laminin	Scaffolds for neural TE	[31]
**PCL**	Soaking in the collagen solution	Scaffolds for dermal substitute	[32]
PCL-CG *	Soaking in BSA-FITC, heparin-FITC, and VEGF	Drug delivery assessment	[33]
**PLGA**	PD, BFP1	Guided bone regeneration	[34]
**PCL**	Hydrophobin, Anti-CD31 antibody	Vascular grafts	[35]
**Cellulose**	Layer-by-layer (LBL) deposition of Au nanoparticles and lysosome	Antibacterial activity	[36]
**PCL**	Ar or O_2_ cold RF plasma; 20–30 W; 5–10 min	Cellular scaffolds	[38]
**PA6**	O_2_ plasma, 100 W, 1–5 min	-	[39]
**Silk fibroin**	CH_4_ plasma	Skin regeneration	[40]
**PLGA**	NH_3_ or O_2_ plasma	Scaffolds for TE	[41]
**PLLA**	O_2_ plasma, 1 min	Scaffolds for TE	[42]
**PCL**	N_2_ + H_2_, NH_3_ + O_2_, and Ar + O_2_ plasma	Scaffolds for cell proliferation	[43]
**PCL**	Air plasma	Vascular grafts	[44]
**PDLG, PLC**	UV photolithography	TE	[45]
**PCL, Gelatin**	Laser ablated pattern	TE	[46]
**PLLA**	Laser ablated pattern	TE	[47]
**PLLA, PCL**	Ultrasound treatment	TE	[48]
**Chitosan**	Ultrasound treatment	Hemostatic material	[49]
**PS, PMMA**	Ultrasound scission	Biomedical use	[50]

* core-shell. PCL—core; glutaraldehyde crosslinked cationized gelatin—shell.

**Table 2:** Methods of chemical modifications of micro- and nanofiber nonwovens and their applications.

Polymer	Method of Modification	Application	Reference
**HA**	a/HCl gas + freezing −20 °C, 20–40 days b/EtOH, HCl aq. 4 °C, 1–2 days	Water-resistant HA membranes	[51]
**PGA**	HCl aq.	Scaffolds for TE	[52]
**PCL**	NaOH aq.	Scaffold for TE	[53]
**PGA**	NaOH aq.	Vascular TE	[54]
**PCL,PHBV**	SBF	Scaffolds for bone TE	[55]
**PLLA**	SBF	Scaffold for bone TE	[56]
**PDLG, PDLG/Gelatin**	a/, b/, or c/	Scaffolds for bone TE	[57]
**Gelatin**	Genipin crosslinking	Scaffolds for neural TE	[58]
**Gelatin**	GA gas	Scaffolds for TE	[59]
**Gelatin type A or B**	GA or EDAC	Scaffolds	[60]

a/ conc. simulated body fluid (SBF); b/ AcOH,CaCl_2_ aq + H_3_PO_4_; c/ (CaCl_2_ 5 min + Na_3_PO_4_ 5 min) several times.

**Table 3:** Methods of physico-chemical modifications of micro- and nanofiber nonwovens and their applications.

Polymer	Method of Modification	Application	Reference
PAN (carbon *)	Heating 250 °C air, calcination 750–1100 °C N_2_	-	[61]
PAN (carbon *)	Heating 280 °C air, 700–1000 °C, Ar, CO_2_	High power supercapacitor	[63]
PAN (carbon *)	Heating 700–1000 °C	-	[64]
TiO_2_ *	Heating and press 120 °C, then 450 °C calcination, O_2_	NO_2_ sensor	[65]
TiO_2_ *, Pt *	Calcination 500 °C, air, 3 h	Hydrazine sensor	[66]
**TiO_2_ nanoparticles**	Calcination	Solar light conversion, hydrogen production	[67]
TiO_2_ *, Pt *	Calcination 510 °C, air	Azo bond reduction	[68]
TiO_2_ *, Ag *	Calcination	Photocatalysis	[69]
SnO_2_ *, NiO *	Calcination	Humidity sensor	[70]
SnO_2_ *, NiO *	Calcination	Formaldehyde sensor	[71]
SnO_2_ *	Calcination	Ethanol sensor	[72]
ZnO *, SnO_2_ *	Calcination	Ethanol sensor	[73]
SnO_2_ *, CuO *	Calcination	H_2_S sensor	[74]
a/SnO_2_ * b/SnO_2_ *, Pd *	Heating + press, calcination a/ 450 °C, b/600 °C	H_2_ and NO_2_ sensor	[75]
SnO_2_ *	Calcination	H_2_ sensor	[76]
NiO *, Pt *	Calcination	Non-enzymatic glucose sensor	[77]
ZrO_2_ *, TiO_2_ *	Calcination	Humidity sensor	[78]
SiO_2_ *, Ag *	Calcination, air 700 °C	Catalysis	[79]
**PGA, PLLA, PLGA**	O_2_ plasma + AA grafting	Scaffolds	[81]
**PCL**	Ar plasma + O_2_ plasma or AA plasma grafting	Scaffolds for bone TE	[82]
**PLLA**	Plasma + CG, CDI grafting	Cartilage tissue engineering, in vivo	[83]
**PCL**	Remote plasma + collagen I	Scaffold for TE	[84]
**PLLA**	O_2_ plasma + RGD peptide, EDAC/sulfo NHS	Osteodoinductive scaffolds	[85]
**PLC**	1,6-(CH_2_)_6_(NH_2_)_2_ aq. 2 min, glutaraldehyde, fibronectin	Esophagus TE	[86]
**PCL**	NaOH aq., EDAC, Matrigel	Neural TE	[87]
**PCL**	UV grafting MMA, EDAC, gelatin	TE	[88]
**PCL + PLGA-b-PEO-NH_2_**	EGS + lysosome	Immobilization of bioactive molecules	[89]

* precursors.

## Abbreviations

AA	acrylic acid
AcOH	acetic acid
aq.	water solution,
BFP1	bone-forming peptide 1
BLG	β -lactoglobulin
BSA	bovine serum albumin
BSA – FITC	bovine serum albumin conjugated with fluorescein isothiocyanate
CDI	carbodiimide
CelluloseNF	cellulose nanofibers
CG	cationized gelatin
conc.	concentrated
EDAC	1-ethyl-3-(3-dimethylaminopropyl) carbodiimide hydrochloride
EGS	ethylene glycol-bis(sulfosuccinimidylsuccinate)
EtOH	ethanol
HA	hyaluronic acid
HAP	hydroxyapatite
Heparin – FITC	heparin conjugated with fluorescein isothiocyanate
HSA	human serum albumin
LBL	layer-by-layer self-assembly technique
PA6	poly(caprolactam), polyamide-6
PAN	poly(acrylonitrile)
PBS	phosphate buffer saline
PCL	poly(ε- caprolactone)
PD	poly(dopamine)
PDLG	poly(D,L-lactide-*co*-glycolide)
PEO	poly(ethylene oxide), poly(ethylene glycol)
PGA	poly(glycolide)
PHBV	poly(hydroxybutyrate-*co*-hydroxyvalerate)
PLC	poly(L-lactide-*co*-caprolactone)
PLGA	poly(lactide-*co-*glycolide)
PLGA-b-PEO-NH2	block copolymer of poly(lactide-*co*-glycolide) with amine-terminated poly(ethylene oxide)
PLLA	poly(L-lactide)
PMMA	poly(methyl methacrylate)
PS	poly(styrene)
PVA	poly(vinyl alcohol)
PVAc	poly(vinyl acetate)
PVP	poly(vinyl pyrrolidone)
RF	radio frequency
RGD	arginylglycylaspartic acid (tripeptide Arg-Gly-Asp)
RhB	rhodamin - B
SBF	simulated body fluid
SBS	styrene-butadiene-styrene triblock copolymer
SF	silk fibroin
SulfoNHS	N-hydroxysulfosuccinimide sodium salt
TE	tissue engineering
VEGF	vascular endothelial growth factor
WP	whey protein
